# Successful weaning from the invasive respiratory support after nusinersen treatment in a child with SMA type 1: A case report

**DOI:** 10.3389/fped.2023.1097063

**Published:** 2023-02-15

**Authors:** Meiling Pan, Jun Shi, Hongjun Miao, Qin Zhang

**Affiliations:** Department of Emergency and Critical Care Medicine, Children’s Hospital of Nanjing Medical University, Nanjing, China

**Keywords:** type 1 SMA, nusinersen, mechanical ventilation, case report, pediatric

## Abstract

**Background:**

Spinal muscular atrophy (SMA) is an autosomal recessive disease, which can be classified into 4 types according to the symptom onset age and the highest physical developmental milestone. Among them, type 1 SMA is the most severe form that affects infants younger than 6 months. Permanent assisted ventilation is usually needed for infants with type 1 SMA before the age of 2 years due to the rapid progression of disease. Nusinersen can improve the motor function of SMA patients, but its effect on respiratory function varies. In the present study, we reported a case of child with type 1 SMA who was successfully weaned from the invasive respiratory support after nusinersen treatment.

**Case presentation:**

A girl aged 6 years and 5 months was admitted for SMA in the Children’s Hospital of Nanjing Medical University for 18 times. She received the first administration of nusinersen in November 2020 at the age of 5 years and 1 month. At the age of 6 years and 1 month following 6 loading doses, we tried to wean the child from the invasive ventilation for non-invasive respiratory support using a nasal mask. At present, the patient shows oxygen saturation (SpO_2_) above 95% without ventilator support during the daytime, and no signs of dyspnea. A non-invasive home ventilator was used at nighttime for the sake of safety. The CHOP INTEND score increased by 11 points from the first loading dose to the sixth. She can now move her limbs against gravity, take in food orally and perform partial vocal function.

**Conclusions:**

We reported a child with type 1 SMA who was successfully weaned from the 2-years invasive ventilation after 6 loading doses, and now only need non-invasive ventilation 12 h per day. It is suggested that even a late nusinersen treatment can improve respiratory and motor function in SMA patients, and wean them from mechanical ventilation, thus improve the quality of life and reduce the medical cost.

## Introduction

Spinal muscular atrophy (SMA) is an autosomal recessive disease characterized by progressive myasthenia and hypotonia resulting from degeneration of motor neurons in the anterior horn cells. In its pathogenesis, survival motor neuron 1 (*SMN1*, OMIM 600354) located on chromosome 5q13.2 undergoes mutation, which further leads to the downregulation of full-length SMN protein. The *SMN2* gene located on the same chromosomal locus is highly homologous to *SMN1*. Due to the differences in bases, most of the proteins encoded by *SMN2* are nonfunctional, with only 10% functional (1). The severity of SMA is closely linked with the *SMN2* copy number. SMA is classified into 4 types, based on the symptom onset age and the highest physical developmental milestone. Among them, type 1 SMA is the most severe form that affects infants younger than 6 months. Permanent assisted ventilation is usually needed for infants with type 1 SMA before the age of 2 years due to the rapid progression of disease. Growing clinical evidence of disease-modifying drug trials and real-world data have shown improved outcomes of SMA patients with a gradually decreasing dependence on airway management. Nusinersen, a synthetic antisense oligonucleotide, targets the pre-mRNA of *SMN2* gene and mediates its splicing, thereby promoting the production of full-length SMN protein. The administration of nusinersen in the early stage is recommended to symptomatic and pre-symptomatic children, especially infants younger than 2 years. However, the benefits of pulmonary rehabilitation remain unclear in old patients or those with severe neuromuscular and pulmonary diseases who are medicated with disease-modifying drugs. Nusinersen is validated to improve the motor function of SMA patients, but its pharmacological effect on respiratory function varies (2, 3). In the present study, we reported a case of child with type 1 SMA who was successfully weaned from the invasive respiratory support after nusinersen treatment at the age of 5 years, because nusinersen has not been marketed in her infancy. Through case report and literature review, we aimed to verify the therapeutic efficacy of nusinersen in improving respiratory function of SMA patients.

## Case description

A girl aged 6 years and 5 months was admitted for SMA in the Children’s Hospital of Nanjing Medical University for 18 times. Clinical data were collected for analysis after obtaining the written informed consent from her guardians. This study was approved by the Institutional Review Board of the Children’s Hospital of Nanjing Medical University (202206118-1). The child was full-term born to cesarean section with a birth weight of 3 kg. Perinatal diseases, and family history of neurological, metabolic or genetic diseases were not reported. Her elder sister was healthy. The child presented progressive limb weakness at post-birth 3 months, which became more serious at 5 months. She was diagnosed as SMA by genetic testing at 7 months. Multiplex ligation-dependent probe amplification (MLPA) data revealed homozygous deletion in exon 7 and 8 of *SMN1*, and 3 copies in exon 7 and 8 of *SMN2*. The child developed a bell-shaped chest prior to genetic therapy. Neurological examination showed systemic hypotonia, grade 2 muscle strength of upper extremities and grade 1 muscle strength of lower extremities, and reduced deep tendon reflexes. She was unable to completely move bilateral arms and legs under gravity, or independently sit and walk. Malnutrition was determined according to her body weight of 11 kg (HAZ: −2.01SD), height of 100 cm (WAZ: −3.64SD), body mass index (BMI) of 11.0 kg/m^2^ (BMI-Z: −3.61SD), and skinfold thickness of 11 mm at the age of 5 years. The child had a pneumonia history of 1–2 times per year before the age of 4 years. She was given respiratory care using a mechanical insufflation-exsufflation (MIE) device at home at the age of 3 years and 10 months, and respiratory support using a non-invasive ventilator at the age of 4 years and 2 months. The child was admitted to the ICU for cough and dyspnea in January 2020 at the age of 4 years and 3 months. Tracheal intubation and mechanical ventilation were performed for severe dyspnea, which was extubated on the 22nd day, and then discharged as condition was improved. However, she was readmitted at 10 days after discharge for dyspnea, and tracheal intubation and mechanical ventilation were performed again. We tried to remove the ventilator twice, but both failed. We recommended tracheotomy, which was refused by her parents. Later, the child was managed by ventilator support at home through nasotracheal intubation. In November 2020 (5 years), the child received the first general anesthesia descending intrathecal injection of nusinersen (5 ml/12 mg), followed by 3 injection of nusinersen on the 14th, 28th and 63rd day. Nusinersen were injected every 4 months after the 4th injection. The child received 8 injection of nusinersen till now (Table 1). Respiratory function of the child was assessed by analyzing the duration of weaning from ventilator support, parameters of mechanical ventilation, and blood gas analysis, length of stay, pulmonary infection and other parameters during nusinersen treatment. In addition, motor function was assessed using the Children’s Hospital of Philadelphia Infant Test of Neuromuscular Disorders (CHOP INTEND) and Hammersmith Infant Neurological Exam-Part 2 (HINE-2) (Table 1). After 5 loading doses of nusinersen treatment, the child could breathe normally for 2 h after withdrawal from mechanical ventilator support. Three months after 6 injections of nusinersen (6 years 1 month), we tried to wean the child from the invasive ventilation for non-invasive respiratory support using a nasal mask. One month later (the age of 6 years and 2 months), the length of non-invasive respiratory support was gradually reduced from 24 h to 15 h per day. At present, the patient shows oxygen saturation (SpO_2_) above 95% without ventilator support during the daytime, and no signs of dyspnea. A non-invasive home ventilator was used at nighttime for the sake of safety. Thankfully, the CHOP INTEND score increased by 11 points from the first loading dose to the sixth. She can now move her limbs against gravity, take in food orally and perform partial vocal function.

**Table 1:** Respiratory function and motor function during nusinersen treatment in the child with type 1 SMA.

Age	5 years	5 years	5 years 1 month	5 years 2 months	5 years 6 months	5 years 11 months	6 years 2 months	6 years 6 months
Loading doses of nusinersen	1	2	3	4	5	6	7	8
Day	D0	D14	D28	D63	D189	D322	D442	D562
Dose	12 mg	12 mg	12 mg	12 mg	12 mg	12 mg	12 mg	12 mg
SpO_2_%	96	100	97	100	99	100	100	100
pH	7.31	7.44	7.41	7.4	7.45	7.4	/	/
PO_2_ (mmHg)	84.5	114.1	90	120.3	113.3	103.3	/	/
PCO_2_ (mmHg)	42.5	34.6	42	38.9	35	25.5	/	/
Duration of ventilator support (h)	24	24	24	24	22	22	15	12
Duration of weaning from ventilator support (h)	0	0	0	0	2	2	9	12
Ventilation mode	SIMV + PSV	SIMV + PSV	SIMV + PSV	PC-SIMV	SIMV + PSV	PC-SIMV	Non-invasive ST mode	Non-invasive ST mode
FiO_2_ (%)	25	21	30	21	21	21	21	21
PIP	18	18	20	20	15	16	16	15
Peak airway pressure (cmH_2_O)	18	15.5	20	15.4	16.5	13.5	16	15
PEEP (cmH_2_O)	4	4	4	4	4	4	5	5
Frequency of ventilator support	25	15	16	20	20	15	25	19
Length of stay (d)	10	4	11	3	10	3	3	3
Pulmonary infection	Upper respiratory tract infection	N.A	Bronchopneumonia	N.A	Bronchopneumonia	N.A	N.A	N.A
CHOP INTEND (left/right)	13/14	18.5/19.5	21.8/22.8	19/18	22/19	24/24	18/18	23/23
RULM (left/right)	2/3	2/3	2/3	2/2	2/2	2/2	2/3	2/2
HINE-2	3	3	3	3	3	3	3	4 (maintained upright all the time)

SMA, spinal muscular atrophy; SpO_2_, oxygen saturation; SIMV, synchronized intermittent mandatory ventilation; PSV, pressure support ventilation; PC-SIMV, pressure controlled synchronized intermittent mandatory ventilation; ST, spontaneous timed; FiO_2_, fraction of inspired oxygen; PIP, peak inspiratory pressure; PEEP, positive end-expiratory pressure; CHOP INTEND, the Children’s Hospital of Philadelphia Infant Test of Neuromuscular Disorders; N.A, not applicable; RULM, revised upper limb module; HINE-2, Hammersmith Infant Neurological Exam-Part.

## Discussion

SMA is mainly treated through up-regulating functional SMN protein. In recent years, drugs for disease-modifying therapies on the market have significantly enhanced the survival of type 1 SMA, and reduced the duration of ventilator support. For treating SMA with at least one copy of the *SMN2* gene, nusinersen is the first antisense oligonucleotide drug approved by the US Food and Drug Administration and the European Medicines Agency (4). It specifically recognizes the pre-mRNA *SMN2* gene and splices it, thus encoding stable functional SMN protein. In February 2019, nusinersen was approved by the China Food and Drug Administration for treating SMA, then has been widely applied to Chinese patients (5). The present study reported a SMA child who was treated by nusinersen at the age of 5, and successfully weaned from 2-year invasive ventilator support after 6 loading doses. She is now managed by non-invasive ventilation 12 h per day. Our experience demonstrates that nusinersen can improve respiratory function in elder children with SMA, and even realize the weaning from ventilation.

Several real-world studies have demonstrated the positive effect of nusinersen on motor function and survival of type 1 SMA patients, but its role in improving respiratory function, especially in older children, remains to be further explored. Pane et al. (6) have followed up 85 type 1 SMA patients treated by nusinersen for 1 year. CHOP INTEND scores were significantly increased in 76% of patients, suggesting the improvement in motor function. However, 20 type 1 SMA patients required longer use of non-invasive ventilation, and tracheostomy was performed in another 2 patients. Only 2 patients showed a reduction in the duration of non-invasive ventilation. A single-center study in Israel followed up 20 type 1 SMA patients for 2 years. They found that nusinersen treatment did not improve respiratory function as expected, and most patients were static in their need for assisted ventilation (7). Sansone VA et al. (8) analyzed clinical data of 118 type 1 SMA patients, and they found that the treatment of nusinersen before the age of 2 years significantly improved the survival without the need of tracheotomy, and the non-invasive ventilation time was shorter than 16 h. It is suggested that nusinersen effectively alleviated respiratory failure. In a relevant study in France, pulmonary function was monitored in 14 type 2 SMA patients, and their maximal static inspiratory pressure, forced vital capacity and esophageal pressure were significantly improved after 6 injections of nusinersen (9). Consistently, Alamla et al. (10) reported that the incidences of invasive ventilation and assisted respiratory support after nusinersen treatment were significantly lower in type 1 SMA children younger than 6 months than in those older than 6 months, suggesting that an early nusinersen treatment can retard the progression of type 1 SMA. Albrechtsen et al. (11) systematically reviewed the efficacy of nusinersen on SMA. They found that an early treatment, especially that prior to symptom onset, brought with better clinical outcomes, and even restored the normal motor function. However, no cases of weaning from invasive respiratory support have been reported yet, and all the treatments with nusinersen begin before the age of 2 years. Ogawa et al. (12) reported a boy with SMA treated by tracheotomy at 75 days and nusinersen at 99 days. After a 6-month nusinersen treatment, he could temporarily breathe by himself without a ventilator for 1.5 h per day. So far, there is only one case of SMA patient who has been successfully weaned from ventilator after nusinersen treatment (13). This boy was subjected to tracheotomy at the age of 3 months and discharged with a home ventilator. Nusinersen treatment was given at the age of 4 months. After the fifth loading dose of nusinersen, he could breathe by himself for 3 h, and 12 h after the sixth dose (at the age of 14 months). At that time, the patient was only given respiratory support during sleep. Consistently, the present case could be temporarily weaned from the ventilation for 2 h after 5 loading doses of nusinersen. The invasive tracheal intubation was extubated following the sixth dose. Notably, the late initiation of nusinersen treatment (the age of 5 years) due to the late marketization in China and high cost, long duration of invasive ventilation (from February 2020 to December 2021), severe thoracic deformity and malnutrition were all unfavorable to the successful weaning from invasive ventilation. In this case, the early manifestations of the child mainly showed a progressive myasthenia and hypotonia.Non-invasive ventilator started until 4 years and 2 months, and then changed to invasive mechanical ventilation due to recurrent lung infection and respiratory failure. Because of the patient has three copy numbers of exons 7 and 8 of SMN 2 gene, the substitution effect of functional SMN protein formation makes the child's disease progress slowly, which is the main factor for the successful withdrawal of IMV. Through this case report, we consider that nusinersen can improve respiratory function and realize weaning from the invasive respiratory support in type 1 SMA patients with thoracic spinal deformity, even the treatment is initiated after the age of 2 years.

Because tracheotomy was rejected by her parents, the present case had depended on tracheal intubation using a nasal mask for 2 years. Therefore, two efforts were taken for weaning from the invasive respiratory support. Nasotracheal intubation was first extubated, and then non-invasive ventilation using a nasal mask was given for 24 h per day. The duration of non-invasive ventilation was progressively reduced at daytime, but maintained at nighttime. Finally, the patient was successfully weaned from the non-invasive ventilation. Tracheostomy has many advantages, including avoidance of oropharyngeal injury, less exposure to sedatives, more comforts, preservation of swallowing and glottic function, safe re-intubation, and easier weaning from mechanical ventilation. However, it is rejected by most of Chinese parents. Tracheotomy for airway management is still recommended in the future for children with long-term mechanical ventilation.

We reported a 6 years and 5 months child with type 1 SMA who was successfully weaned from the 2-years invasive ventilation after 6 loading doses, and now only need non-invasive ventilation 12 h per day. It is suggested that even a late nusinersen treatment can improve respiratory and motor function in SMA patients, and wean them from mechanical ventilation, thus improve the quality of life and reduce the medical cost.

## Data Availability

The raw data supporting the conclusions of this article will be made available by the authors, without undue reservation.
